# Crystallographic Anisotropies in α‑SnWO_4_ Photoelectrodes and Their Effects on Electronic Properties and Photoelectrochemical Performances

**DOI:** 10.1021/acsami.6c04445

**Published:** 2026-05-16

**Authors:** Ronen Gottesman, Erwin Fernandez, Rene Schwiddessen, Daniel Abou-Ras, Doron Azulay, Oded Millo, Roel van de Krol

**Affiliations:** 1 The Institute of Chemistry, 450737 The Hebrew University of Jerusalem, Edmond J. Safra Campus, Givat Ram, Jerusalem 9190401, Israel; 2 The Center for Nanoscience and Nanotechnology, 450737 The Hebrew University of Jerusalem, Edmond J. Safra Campus, Givat Ram, Jerusalem 9190401, Israel; 3 Institute for Solar Fuels, 28340 Helmholtz-Zentrum Berlin für Materialien und Energie GmbH, Hahn-Meitner-Platz 1, Berlin 14109, Germany; 4 Department of Structure and Dynamics of Energy Materials, 28340 Helmholtz-Zentrum Berlin für Materialien und Energie GmbH, Hahn-Meitner-Platz 1, Berlin 14109, Germany; 5 The Racah Institute of Physics, 450737 The Hebrew University of Jerusalem, Edmond J. Safra Campus, Givat Ram, Jerusalem 9190401, Israel; 6 Department of Physics, Azrieli College of Engineering, Jerusalem 9103501, Israel; 7 Institut für Chemie, Technische Universität Berlin, Straße des 17. Juni 124, Berlin 10623, Germany

**Keywords:** α-SnWO_4_, photoelectrochemical water splitting, metal oxide photoelectrodes, rapid thermal processing, crystallographic orientation, anisotropic charge transport, band edge alignment, percolation networks

## Abstract

Multinary metal oxide photoelectrodes remain fundamentally limited by poor charge transport despite theoretical promise for solar fuel production. *α*-SnWO_4_ exemplifies this challenge: while density functional theory predicts highly anisotropic charge transport with orientation-dependent band-edge positions, synthetic barriers to achieving phase-pure films with controlled crystallographic orientation have prevented its exploitation. Here, we demonstrate that rapid thermal processing (RTP) of pulsed-laser-deposited films overcomes these synthetic limitations, creating percolation networks of co-oriented grains. Multiscale characterization reveals that aligned crystallographic orientations produce well-aligned band edges, lowering contact potential difference by 0.35 eV and enhancing the local conductivity by more than 2 orders of magnitude compared to furnace heating (FH). These results directly correlate enhanced transport properties with previously reported improved photoelectrochemical performance of the RTP-treated films compared to those treated by FH and suggest a microscopic mechanism for this improvement. Our findings establish that controlling grain orientation connectivity, not simply grain size, provides a scalable pathway for exploiting anisotropic transport in multinary metal oxide photoelectrodes, directly linking the microstructure to the enhanced charge transport required for practical solar fuel devices.

## Graphical abstract

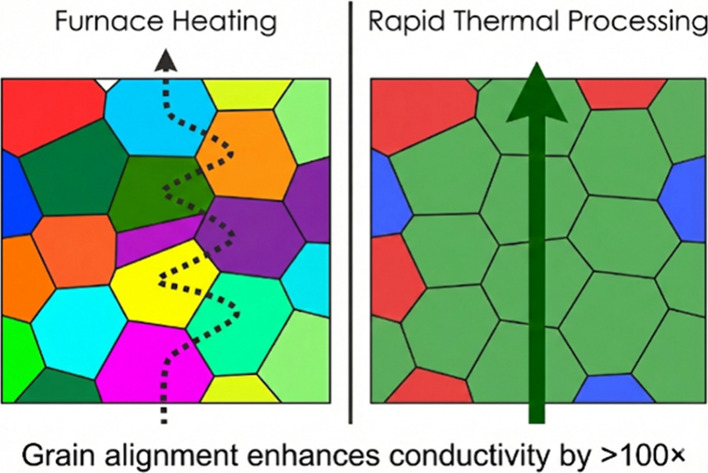

## Introduction

To achieve the global target of net-zero emissions by 2050, decarbonizing our energy systems requires accelerating the development of sustainable chemical processes powered by renewable energy sources.^1–3^ While various approaches exist for renewable chemical synthesis, photoelectrochemical (PEC) cells offer a transformative approach by directly converting solar energy into valuable chemicals and fuels.^4–11^ Metal oxides dominate PEC research due to their chemical stability and earth abundance, but their performance is fundamentally limited by slow charge transport arising from carrier localization.^12–19^

*α*-SnWO_4_ has emerged as one of the most promising metal-oxide photoanodes, combining attractive theoretical properties with significant synthetic challenges that have hindered its development.^20–25^ This *n*-type semiconductor comprises earth-abundant elements and has an indirect bandgap of ∼1.9 eV. This bandgap enables a theoretical maximum photocurrent density of ∼17 mA/cm^2^ under AM1.5 irradiation, assuming all photons with energies larger than the bandgap are absorbed and collected. Its valence and conduction band edge positions straddle the water oxidation and reduction potentials, making it suitable for a range of electrochemical processes. It has a reported mobility of 0.2 cm^2^/V·s, which is quite similar to that of BiVO_4_, one of the best-performing metal oxides for PEC devices.^26^ Due to its narrower bandgap, *α*-SnWO_4_ can theoretically reach efficiencies more than twice that of BiVO_4_ (having a ∼2.4 eV bandgap, yielding a theoretical maximum photocurrent density of ∼7.5 mA/cm^2^).^25^

However, the progress and development of *α*-SnWO_4_ are confronted by two limiting factors: First, there are synthetic challenges to achieve phase-pure *α*-SnWO_4_ with minimal structural defects, mainly because the tendency of Sn^2+^ to oxidize to Sn^4+^ and/or to disproportionate: 2Sn^2+^ → Sn^4+^ + Sn^0^.^25,27–30^ To prevent the formation of impurity phases such as the reported SnW_3_O_9_, Sn_0.23_WO_3_, Sn_0.11_WO_3_, and SnO_2_ phases, growing pure *α*-SnWO_4_ requires careful heating (or postannealing in some synthetic methods) in a narrow temperature range of ∼500 to 550 °C in an inert atmosphere (i.e., N_2_ or Ar). Second, the reported carrier diffusion length (*L*_D_ ∼10 to 100 nm, measured in polycrystalline films) is 10^3^ times smaller than the photon penetration depth.^17,25,29^

Hybrid density functional theory calculations indicate that *α*-SnWO_4_ exhibits anisotropic charge-transport characteristics that influence its electrochemical performance in various reactions, including H_2_ and O_2_ evolution.^31–33^ However, in-depth experimental studies on *α*-SnWO_4_ with different preferred crystallographic orientations have yet to be reported, presumably due to synthetic challenges (see above). A recent review summarizes the progress in the development of *α*-SnWO_4_ photoanodes over the past several years,^25^ including various synthesis approaches to enhance their structural and electronic quality, efficiency, and stability, such as conversion from WO_3_, magnetron sputtering, and pulsed laser deposition (PLD).^25^ Despite showing an impressive two-orders-of-magnitude improvement in the photoelectrochemical performance, studying *α*-SnWO_4_ thin-film photoanodes remains underdeveloped, especially its anisotropic charge transport characteristics, calling for suitable strategies to overcome its significant synthetic limitations. RTP is one such strategy, in use in semiconductor manufacturing since the 1980s, with recent extensions to commercially scalable flash photonic annealing further expanding its applicability to metal-oxide photoelectrodes.^27,34,35^

In a previous study,^32^ we deposited amorphous SnWO_4_ films on glass-based SnO_2_:F substrates (FTO) via PLD and postannealed them at 550 °C using either rapid thermal processing (RTP) for 1 to 8 min or in a tube furnace (furnace heating FH, 2 h). While both methods produced single-phase *α*-SnWO_4_, RTP treatment yielded films with enhanced crystallinity and distinct crystallographic orientations compared to FH treatment. Photoelectrochemical measurements revealed that RTP-treated photoelectrodes exhibited photocurrents up to ∼0.95 mA/cm^2^ for sulfite oxidation, approximately 70% higher than FH-treated samples (∼0.57 mA/cm^2^). This performance enhancement was attributed to improved crystallinity, electronic structure modifications, and crystallographic orientation effects. Given the distinct performance characteristics and the representative nature of their responses, we selected the 2 h FH-treated photoelectrode and the optimal 8 min RTP-treated photoelectrode for further detailed investigation in this study. The present study, therefore, focuses on identifying the structural and electronic origins of the performance differences between the two types of thermal treatments reported in (Gottesman et al.),^32^ rather than on photoelectrochemical characterization, which was the subject of that earlier work.

## Results and Discussion

In the present work, we further investigated the structural and electronic properties of 2 h FH-treated and 8 min RTP-treated *α*-SnWO_4_ films, prepared by PLD (Figure 1), by utilizing electron backscatter diffraction in scanning electron microscopy, X-ray diffraction texture analysis, and various atomic force microscopy (AFM) modes: Kelvin probe force microscopy (KPFM), conductive-AFM, and tapping mode imaging for surface morphology. To investigate in more detail the differences between the two film types, we first characterized the thermal profiles and the resulting crystallographic orientations for both treatments.

**Figure 1:**
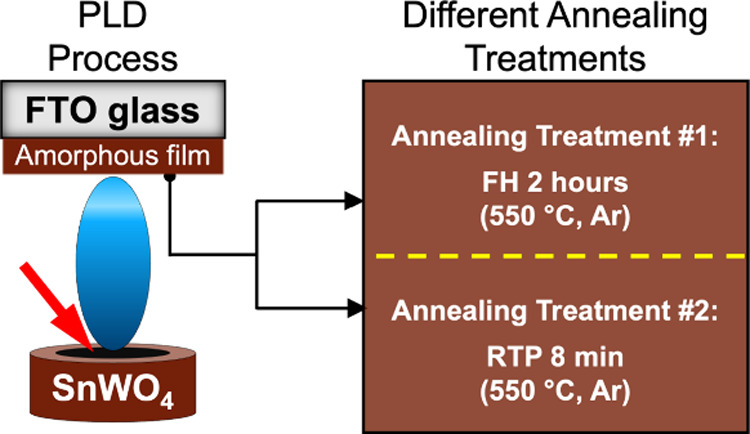
Illustration of the growth method of SnWO_4_ films via a PLD process. The as-deposited amorphous SnWO_4_ film is divided into two identical samples, which are then subjected to two different annealing treatments: furnace heating (FH, 2 h, 550 °C, Ar) and rapid thermal processing (RTP, 8 min, 550 °C, Ar).

### Crystallographic Properties

The thermal processing protocols and their crystallographic outcomes are presented in Figure 2a and Figure 2b, respectively, showing the temperature profiles over time during annealing and the corresponding X-ray diffractograms of the SnWO_4_ thin films. The heating and cooling phases were short for RTP, with a holding duration at 550 °C for only 8 min. In contrast, the heating and cooling phases are much longer in the FH process, with a 2 h holding time at 550 °C. As shown in Figure 2b, the RTP and FH treatments resulted in different crystallographic orientations in the *α*-SnWO_4_ polycrystalline films. The peak at 2θ ≈ 29°, marked with an asterisk, corresponds to the (031) reflection of *α*-SnWO_4_. The XRD data were acquired from samples prepared independently for this study under identical conditions to those reported in (Gottesman et al.),^32^ confirming the reproducibility of the crystallographic textures.

**Figure 2:**
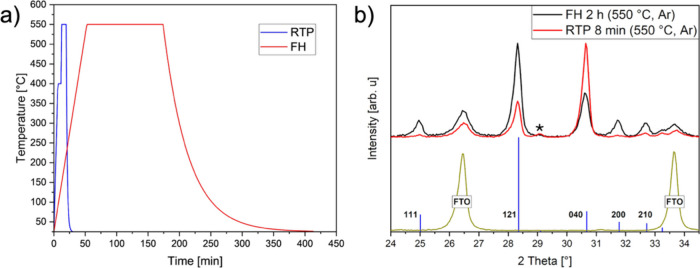
(a) The temperature vs time profiles during the annealing of the SnWO_4_ thin films differ for RTP and FH. (b) X-ray diffractograms of the FH-treated and 8 min RTP-treated films in the 2θ range of 24–34.5°, exhibiting the 111, 121, 040, 200, and 210 crystallographic orientations. The asterisk (*) marks the (031) reflection of *α*-SnWO_4_, a low-intensity reflection present in the full calculated diffraction pattern (PDF 04-011-0013).

To analyze and map the crystallographic orientation, grain structure, and grain boundary characteristics at the micro- to nanoscale, we conducted electron backscatter diffraction (EBSD) measurements on both samples.

The resulting orientation distributions from EBSD are shown in Figure 3 (for the in-plane directions *X* and *Y*, as well as the direction perpendicular to the substrate, *Z*). The EBSD maps reveal domains with similar colors, indicating similar local crystal orientations, as seen in both films. The crystallographic orientation analysis reveals additional distinctions between the two treatments. In the direction perpendicular to the substrate (*Z*), the SnWO_4_ thin films grown by both procedures exhibit a strong preference for grain orientations perpendicular to {010}. A preferred orientation parallel to ⟨010⟩ in the *Z* direction is also found for the FTO substrate, as highlighted by the green false-color in the EBSD map (Figure 3g). No preferred orientations are detected in the in-plane orientation distributions (*X* and*Y*), indicating a fiber texture in the SnWO_4_ and FTO thin films.

**Figure 3:**
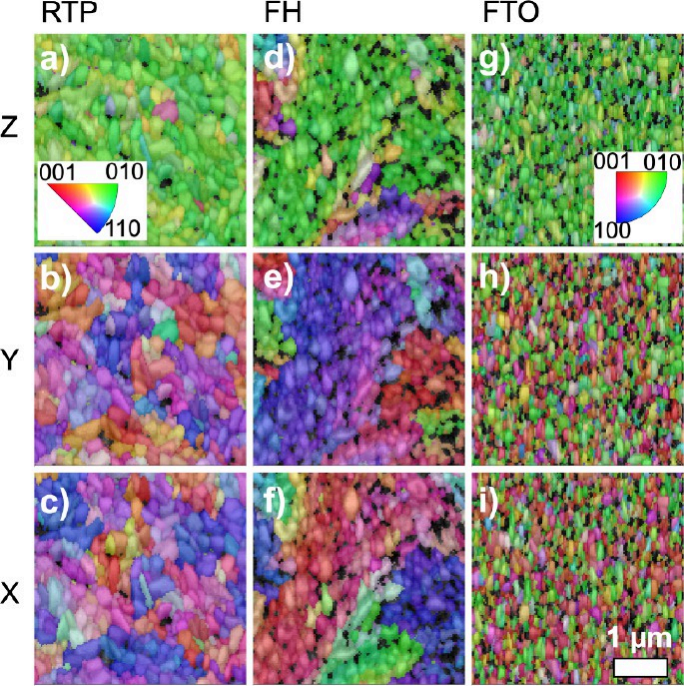
Electron backscatter diffraction (EBSD) orientation maps of *α*-SnWO_4_ thin films grown on FTO substrates and of bare substrates. (a–c) FH-treated film showing orientation distributions in *X*, *Y*, and *Z* directions. (d–f) 8 min RTP-treated film showing orientation distributions in *X*, *Y*, and *Z* directions. (g–i) Bare FTO substrates. The color coding represents different crystallographic orientations, with domains of similar colors indicating regions with similar local crystal orientations. The inset of panel (a) displays the color coding, and the scale bar in panel (i) indicates the dimensions of the measurement area.

Indeed, fiber textures in SnWO_4_ and SnO_2_ thin films are clearly visible in the pole figures extracted from the EBSD data sets (Figure 4). The degree of preferred orientation (expressed as multiples of the intensity of the random distribution) is similar for FH-grown SnWO_4_ films (maximum of 9) and RTP-grown SnWO_4_ films (maximum of 7). An analysis of the corresponding crystal facets of the SnWO_4_ and SnO_2_ structures that grew on top of one another revealed an orientation relationship between the two materials. The Sn sublattice forms rhombi (highlighted in Figure 4d,e by green lines) with side lengths of 0.37 nm in the tetragonal SnO_2_ crystal structure. To obtain the SnWO_4_ structure, W atoms have to be inserted into these rhombi, leading to a substantial distortion in the horizontal direction (Sn–Sn bonding length increases from 0.32 to 0.56 nm). In comparison, the interatomic distances in the vertical direction remain the same (about 0.5 nm). Due to this substantial distortion, strong strain fields are expected at the SnWO_4_/SnO_2_ interface, which the material system can compensate at least in part via elemental interdiffusion (i.e., W) across this interface.

**Figure 4:**
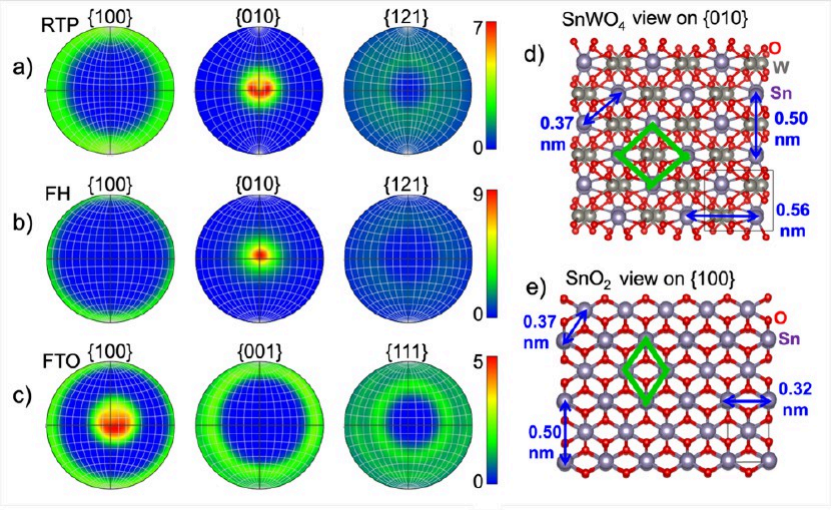
Pole figures extracted from EBSD data sets and structural models of SnWO_4_ and SnO_2_. (a, b) Pole figures for FH-treated and RTP-treated SnWO_4_ films, respectively, with intensity values given in multiples of random distribution. (c) Pole figure of the underlying FTO (SnO_2_) substrate. (d, e) Crystal structure models of SnWO_4_ and SnO_2_ with Sn sublattice rhombi highlighted in green lines. Interatomic distances are shown for key structural features (generated using the VESTA software).^36^

### Electrical Properties

We shall now turn to the discussion of the electrical properties of the RTP- and FH-treated films, starting with the contact potential difference (CPD) as measured by KPFM using a Pt-coated Si cantilever. The work-function of the tip was calibrated with respect to Au and Al films and was found to be 4.9 ± 0.1 eV. Figure 5 presents CPD maps along with the corresponding topographic maps for the RTP (Figure 5a,b) and FH (Figure 5d,e) treated films, where the histograms of the CPD values are shown to the right (Figure 5c and Figure 5f, respectively). Both films are approximately 100 nm thick (Gottesman et al.).^32^ There are two notable differences between the two data sets. First, while the topographic image of the RTP-treated film exhibits round-shaped grains, that of the FH-treated sample shows elongated grains. Also, the surface corrugation of the latter sample is somewhat larger than that of the former, with RMS roughness values of 19 ± 1 nm (RTP) and 22 ± 3 nm (FH). The elongated grain morphology and increased surface corrugation of the FH-treated film are consistent with the longer thermal budget of the FH process, which provides more time for grain growth and microstructural coarsening compared to RTP. Notably, the physical grain size visible in the AFM topography of the FH-treated film is substantially larger than the crystallographic domain size resolved by EBSD, suggesting that individual FH grains contain multiple subgrain domains with slightly differing orientations. This internal crystallographic complexity may contribute additional potential barriers to charge transport within the FH film, beyond those arising from intergrain misorientation. Second, the CPD values (with respect to the Pt-coated tip) measured on the FH-treated sample are larger than those of the RTP one, as clearly manifested by the histograms depicting mean values of ∼0.6 V (FH) and ∼0.25 V (RTP), meaning the work-function of the FH-treated sample is smaller than that of the RTP-treated one by ∼0.35 eV. CPD measurements acquired from multiple scan areas on each sample confirm the consistency of these values, yielding 0.256 ± 0.007 V (RTP) and 0.615 ± 0.008 V (FH) (see Supporting Information, Figure S1).

**Figure 5:**
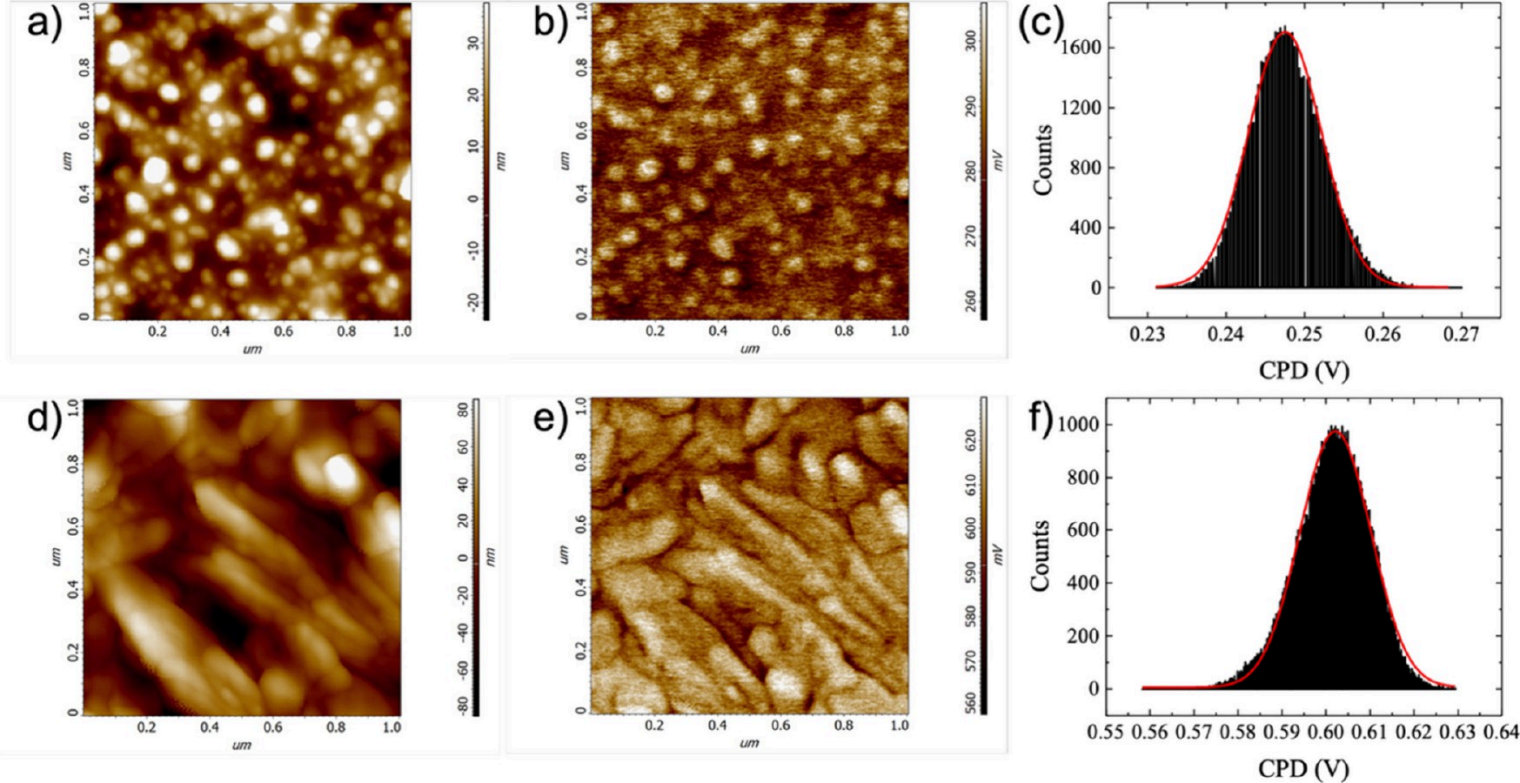
CPD measurements on SnWO_4_ films. (a, d) Topographic maps of the RTP-treated (a) and FH-treated (d) films. (b, e) CPD maps acquired with a Pt-coated Si tip (work function of ∼4.9 eV) from the RTP- (b) and FH-treated films (e). (c, f) Histograms of the CPD values, RTP (c) and FH (f).

The electrical transport properties of the FH and RTP-treated films also differ significantly, as demonstrated by the conductive-AFM data presented in Figure 6. Figure 6a (RTP) and Figure 6d (FH) display representative local current–voltage (*I*–*V*) characteristics measured between the Pt-coated AFM tip and the FTO glass underlying the SnWO_4_ film (*V* is the bias applied to the FTO). Evidently, the RTP-treated sample is by far more conductive compared with its FH-treated counterpart. The current measured on the RTP-treated sample reaches the preamp saturation value of 25 nA already at sample biases of ±0.2 V, while that of the FH-treated film is far from this value, even at *V* = −8 V. Moreover, the FH-treated film manifests a pronounced rectifying behavior characteristic of Schottky barrier formation between the Pt-coated tip and the film. This can be explained by the fact that the surface work-function of the (*n*-type) SnWO_4_ film is approximately 0.6 eV lower than that of the Pt-coated tip (see Figure 5f), facilitating the formation of such a barrier much larger than that with the RTP-treated sample, where the difference is much smaller, ∼0.24 eV (Figure 5c). The current maps presented in Figure 6c (RTP) and Figure 6f (FH) further manifest the higher conductivity of the RTP sample. Note that the current map in Figure 6f was measured at *V* = −6 V, much higher (in magnitude) than the −0.3 V used for the RTP-treated sample in Figure 6c. Furthermore, the current maps, when correlated with the corresponding topographic images, reveal that the (small) current detected under negative bias voltages for the FH-treated samples flows mainly through a network of grain boundaries (Figure 6e,f)—bypassing the potential barriers between misoriented adjacent grains (see below). In contrast, the current maps of the RTP-treated samples display a more homogeneous (saturated) current distribution across the grains (Figure 6b,c).

**Figure 6:**
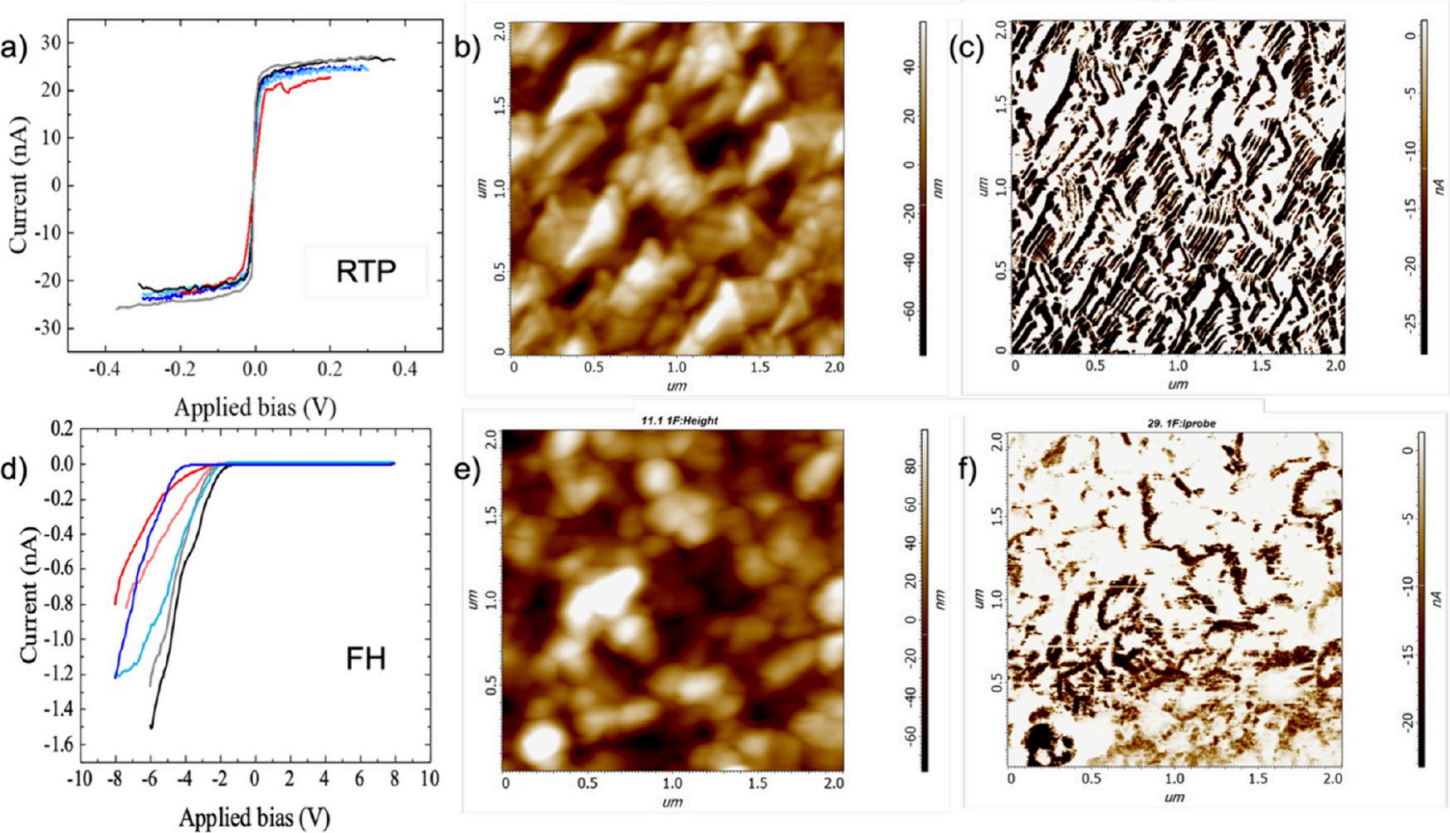
Conductive-AFM measurements on SnWO_4_ films. (a) Representative local *I*–*V* characteristics measured at different locations along the RTP-treated film. (b) Topography map of the RTP-treated film. (c) Current map of the RTP-treated film measured under a sample bias of −0.3 V. (d) Representative local *I*–*V* characteristics measured at different locations along the FH-treated film. (e) Topography map of the FH-treated film. (f) Current map of the FH-treated film measured under a sample bias of −6 V.

The significant difference in conductivity in the RTP- and FH-treated samples can be further explained by the relatively large variations in energy gap and the energetic alignment of the conduction and valence bands across different crystallographic orientations in *α*-SnWO_4_ (Table 1),^31–33^ which can form potential barriers between adjacent grains that hinder electron transport. Therefore, two possible conductive networks can exist in this system: a high-conductivity network formed by co-oriented grains, where the band edges align, and there are small or no potential barriers; and a low-conductivity network, where large potential barriers between misoriented grains impede electron transport.

**Table 1:** Band Energy Levels of Different Crystallographic Orientations in *α*-SnWO_4_ ^a^

	(111)	(121)	(040)	(200)	(210)
CBM	–0.7	–0.3	+0.5	–0.9	–0.9
VBM	+1.2	+1.2	+1.6	+1.2	+1.5

a Conduction band minimum (CBM) and valence band maximum (VBM) values are provided in eV relative to the vacuum level for the (111), (121), (040), (200), and (210) lattice planes. Data compiled from hybrid density functional theory calculations reported in refs 31–33 .

It is important to note that the transport mechanism within individual grains, commonly described as small polaron hopping in *α*-SnWO_4_ and related transition-metal oxides,^37,38^ is not altered by the choice of thermal processing. What differs between RTP- and FH-treated films is the energy landscape that charge carriers encounter at grain boundaries. As seen in Figure 7, a percolation network of co-oriented grains exists in the RTP-treated samples, where band edges align across adjacent grains and intergrain potential barriers are minimal or absent. In contrast, such a percolation network is absent in the FH-treated samples, where misoriented grains create substantial potential barriers at their boundaries, resulting in significantly lower conductivity compared with the RTP samples and transport takes place mainly along grain (crystallite) boundaries, as demonstrated in Figure 6f. Charge carriers therefore preferentially move through contiguous regions of co-oriented grains with low barrier heights, effectively bypassing high-barrier interfaces. This yields an inhomogeneous transport network in which the macroscopic conductivity is controlled by the existence of continuous low-energy percolation paths. Moreover, boundaries between misoriented grains are likely to have a high concentration of charged defects, creating additional potential barriers and scattering centers, consistent with the mechanism proposed by Warren et al. for hematite photoanodes.^39^ Our combined electron microscopy and AFM results suggest that the RTP treatment increases the fraction and connectivity of co-oriented grains in *α*-SnWO_4_ and thus improves the macroscopic conductivity of the film.

**Figure 7:**
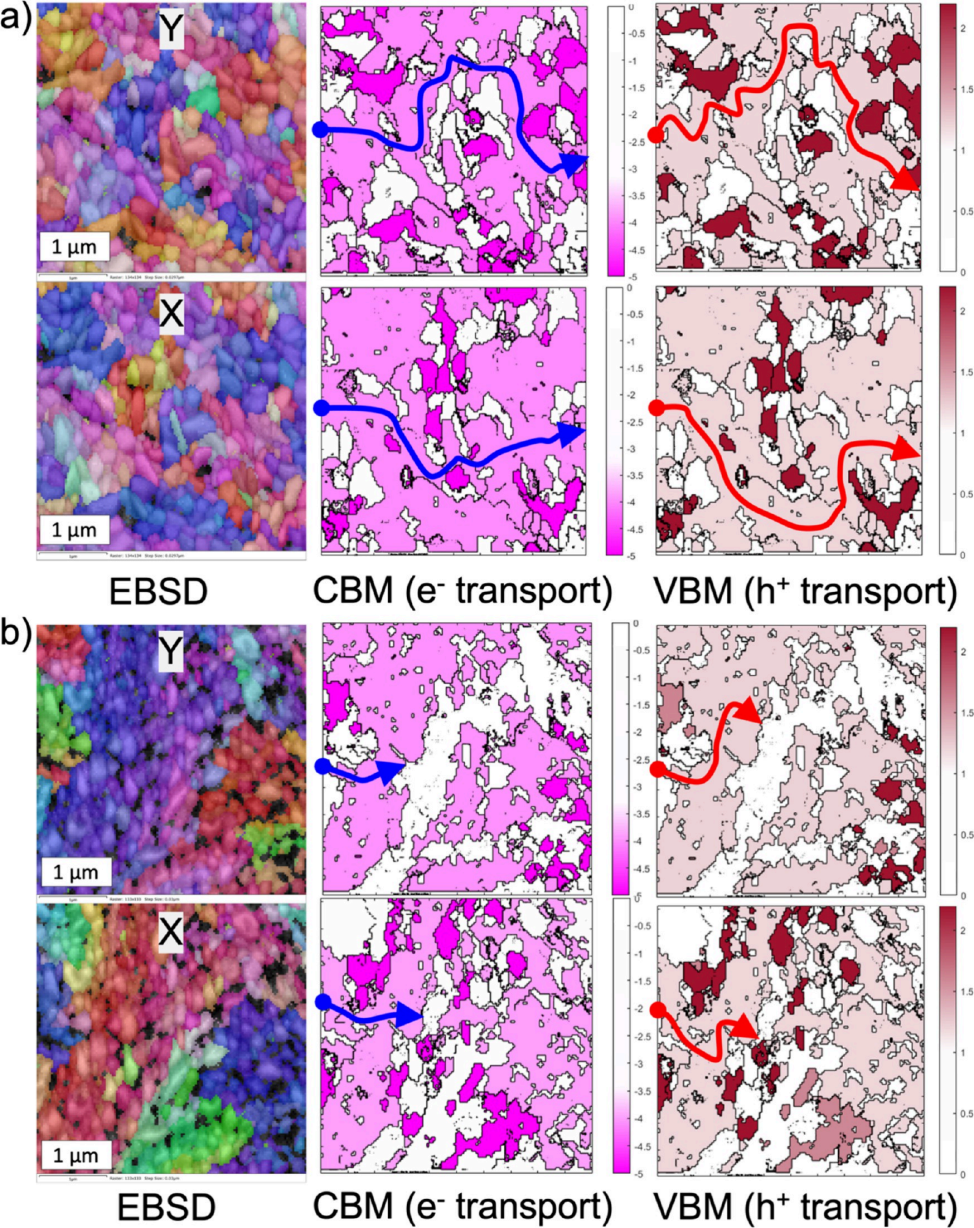
Suggested mechanism for increased conductivity in RTP-treated films (a) compared to FH-treated films (b). Each panel displays the measured EBSD data in the *X* and *Y* directions, along with corresponding CBM and VBM energy maps constructed from the EBSD data and the energy values in Table 1. The arrows represent a random 4 μm-long charge-carrier transport pathway through the polycrystalline film, illustrating how carriers encounter different energy landscapes. In the CBM maps, electrons (e^–^) move from higher energy (brighter) to lower energy (darker) grains, while in the VBM maps, holes (h^+^) move from lower energy (darker) to higher energy (brighter) grains. Different color scales are used for the CBM and VBM maps to highlight the energy variations within each band.

## Conclusions

Rapid thermal processing fundamentally improves intergrain charge transport in *α*-SnWO_4_ photoelectrodes, providing a mechanistic explanation for the enhanced photoelectrochemical performance reported in Gottesman et al. RTP achieves this by creating percolation networks of co-oriented grains with aligned band edges, reducing contact potential differences by 0.35 eV and enhancing local conductivity by over 2 orders of magnitude. These transport improvements establish that grain orientation connectivity, rather than grain size alone, governs charge transport in these films, providing direct evidence for the role of band edge alignment in reducing potential barriers between adjacent grains. Exploiting crystallographic anisotropies in *α*-SnWO_4_ offers a critical pathway for enhancing charge transport beyond defect minimization alone. Aligned grain networks with matching band edge positions create superior transport pathways by minimizing potential barriers between adjacent grains in polycrystalline films. This approach leverages predicted orientation-dependent band-edge positions in *α*-SnWO_4_, where theoretical calculations indicate substantial energy variations across different crystallographic facets. Microstructural engineering through orientation control thus addresses transport limitations that defect reduction alone cannot overcome, even in high-quality films.

However, predictive relationships between processing parameters and the degree of orientation control achievable remain to be established. Moving forward, systematic investigation of RTP parameters and substrate textures could reveal predictive relationships for controlling grain orientation in *α*-SnWO_4_ and related materials. Macroscopic transport measurements on insulating substrates, including temperature-dependent resistivity, could further clarify the relative contributions of intragrain polaron hopping and intergrain barrier resistance, though such studies must account for substrate-dependent differences in film microstructure. The significance of grain orientation effects is expected to be particularly pronounced in multinary metal oxides, which possess lower crystal symmetry and more degrees of freedom in orientation and growth compared to binary oxides, amplifying the electronic consequences of grain misorientation. Translation of these orientation-controlled principles to other anisotropic multinary oxides could establish whether exploiting crystallographic anisotropy represents a general strategy for enhancing charge transport in polycrystalline photoelectrodes. Implementing these fundamental structure–property insights into complete photoelectrochemical devices requires integration with optimized cocatalysts and interface engineering that could be characterized by relevant macroscopic techniques, such as electrochemical impedance spectroscopy.^40^ The available scalability of RTP processing positions this approach as a major candidate for such device integration, where efficient carrier transport remains critical for achieving practical solar-to-fuel conversion efficiencies.

## Experimental Section

### Film Deposition

Films were deposited using a PLD system (PREVAC, Poland) by ablating with a KrF-Excimer laser (248 nm, LPXPro 210, COHERENT) a commercial SnWO_4_ target (99.99%, METALLIC FLEX). The target-to-substrate distance was 60 mm, and all substrates were FTO-coated glass (TEC 7, Pilkington). Substrates were cleaned for 10 min in each of the following solutions: 1 vol % Triton X-100 (Sigma-Aldrich) in deionized water, and ethanol (Sigma-Aldrich). Films were deposited at room temperature under a background pressure of 1 × 10^–4^ mbar with a laser fluence of 2 J/cm^2^.

### Furnace Heating

Conventional furnace heating of the SnWO_4_ films was performed at 550 °C in Ar at a heating rate of 10 °C/min, with a 2 h hold time.

### Rapid Thermal Processing

Rapid thermal processing was performed using a Rapid Thermal Processor (AS-One 100, ANNEALSYS). In a typical RTP procedure, a sample is placed on a SiC wafer, which serves as a susceptor, with an optical pyrometer monitoring the SiC’s temperature. Additionally, the samples’ surface temperatures were monitored with a thermocouple attached to the surface using an indium contact. The susceptor with the sample placed on top is slowly heated to 400 °C (rate = 1 K/s) and then maintained at that temperature for 5 min. Next, the temperature is rapidly increased to 550 °C (rate = 10 K/s) in Ar atmosphere (1 atm), and maintained for 8 min.

### X-ray Diffraction

X-ray diffraction measurements were performed using a Bruker D8 diffractometer with Cu Kα radiation. Measurements were carried out in grazing-incidence geometry (angle of incidence = 2°) with a step size of 0.04° and a step duration of 6 s. The data were normalized after background subtraction, without additional averaging or noise reduction.

### Electron Backscatter Diffraction (EBSD)

EBSD data sets were acquired using a Zeiss UltraPlus scanning electron microscope equipped with an Oxford Instruments Symmetry EBSD detector at 15 keV and 6 nA. The software tool AZtec was used for the acquisition and evaluation of the EBSD data sets.

### AFM Characterization

Atomic force microscopy (AFM) measurements were performed to investigate the surface morphology and local electrical properties of the films. Conductive AFM (c-AFM) measurements were carried out using Pt-coated Si contact cantilevers with a nominal spring constant of ∼0.18 N/m, enabling simultaneous acquisition of current maps together with topographical data. Kelvin probe force microscopy (KPFM) measurements were conducted using Pt-coated Si noncontact cantilevers with a nominal spring constant of ∼2 N/m and a typical resonance frequency of ∼130 kHz. The KPFM scans were acquired using a two-pass configuration: in the first pass, the surface topography was recorded in tapping mode while the surface potential (contact potential difference) was measured in the second pass, along the previously acquired topographic profile.

## Supplementary Material



## Notes

CPD distributions acquired from multiple scan areas on RTP-treated and FH-treated *α*-SnWO_4_ films (PDF)

## References

[1] Huo J., Wang Z., Oberschelp C., Guillén-Gosálbez G., Hellweg S. (2023). Net-Zero Transition of the Global Chemical Industry
with CO2-Feedstock by 2050: Feasible yet Challenging. Green Chem..

[2] Maka A. O. M., Ghalut T., Elsaye E. (2024). The Pathway towards Decarbonisation
and Net-Zero Emissions by 2050: The Role of Solar Energy Technology. Green Technologies and Sustainability.

[3] Meys R., Kätelhön A., Bachmann M., Winter B., Zibunas C., Suh S., Bardow A. (2021). Achieving Net-Zero
Greenhouse Gas Emission Plastics by a Circular Carbon Economy. Science (1979)..

[4] Khan B., Faheem M. B., Peramaiah K., Nie J., Huang H., Li Z., Liu C., Huang K. W., He J. H. (2024). Unassisted Photoelectrochemical
CO2-to-Liquid Fuel Splitting over 12% Solar Conversion Efficiency. Nat. Commun..

[5] Han C., Wang K. (2025). Recent Advances in Photoelectrochemical Synthesis of
Nitrogen-Containing
Solar Fuels and Chemicals. Energy Fuels.

[6] Li S., Liu H., Chen G., Wu L. Z., Zhang T. (2025). Paired Chemical Upgrading
in Photoelectrochemical Cells. JACS Au.

[7] Sari F. N. I., Chuang P. C., Huang S. C., Lin C. Y., Lai Y. H. (2025). Photoelectrochemical
Valorisation of Organic Waste for the Cogeneration of Solar Fuels
and Value-Added Chemicals. Chemical Science.

[8] Xi Z., Liu M. (2025). Advancing Photoelectrochemical
Systems for Sustainable Energy and
Chemical Production: Challenges and Opportunities. npj Materials Sustainability.

[9] Fu H., Ioka D., Pan Z. (2026). Transition-Metal-Doped Perovskite
Oxide Materials: A Key Pathway for Photocatalytic Water Splitting
Under Visible-Light Irradiation. ACS Catal..

[10] Zhang Y., Duan Y., Wang Q., Gu Y., Li Y., Dong F. (2026). Structural Engineering Strategies
of Aluminum-Based Photocatalysts
for Energy and Environmental Applications. ACS
Catal..

[11] Mamun A. A., Chowdhury A. H., Billah A., Karim J., Hussain A. O., Rahman F., Talukder M. A. (2025). Advancing Transition Metal Oxide
Photoelectrodes for Efficient Solar-Driven Hydrogen Generation: Strategies
and Insights. Adv. Energy Mater..

[12] Jiang C., Moniz S. J. A., Wang A., Zhang T., Tang J. (2017). Photoelectrochemical
Devices for Solar Water Splitting-Materials and Challenges. Chem. Soc. Rev..

[13] Mohd
Raub A. A., Bahru R., Mohd Nashruddin S. N. A., Yunas J. (2024). Advances of Nanostructured Metal Oxide as Photoanode
in Photoelectrochemical (PEC) Water Splitting Application. Heliyon.

[14] Li C., He J., Xiao Y., Li Y., Delaunay J. J. (2020). Earth-Abundant Cu-Based
Metal Oxide Photocathodes for Photoelectrochemical Water Splitting. Energy Environ. Sci..

[15] Abdi, F. F. ; Berglund, S. P. ; van de Krol, R. Multinary Metal Oxide Photoelectrodes. In Photoelectrochemical Solar Fuel Production: From Basic Principles to Advanced Devices; Giménez, S. , Bisquert, J. , Eds.; Springer International Publishing, 2016; pp 355–391. 10.1007/978-3-319-29641-8.

[16] Abdi F. F., Berglund S. P. (2017). Recent Developments in Complex Metal
Oxide Photoelectrodes. J. Phys. D Appl. Phys..

[17] Schleuning M., Kölbach M., Ahmet I., Präg R., Gottesman R., Gunder R., Zhang M., Wargulski D. R., Abou-Ras D., Grave D. A., Abdi F. F., van de
Krol R., Schwarzburg K., Eichberger R., Friedrich D., Hempel H. (2023). Carrier Localization on the Nanometer-Scale Limits
Transport in Metal Oxide Photoabsorbers. Adv.
Funct. Mater..

[18] Shor
Peled S., Miriyala K., Grave D. A. (2026). Disentangling Structural
and Electronic Contributions to Photogenerated Mobile Charge Carrier
Yield and Transport in Fe2O3 Polymorphs. ACS
Appl. Mater. Interfaces.

[19] Saini R. K., Miriyala K., Chernykh D., Engel Y., Rashkovskiy A., Grave D. A. (2026). Intrinsic Charge-Carrier
Transport Limitations in ZnFe2O4
Revealed by Time-Resolved Microwave Conductivity. J. Phys. Chem. Lett..

[20] Ke J., Adnan Younis M., Kong Y., Zhou H., Liu J., Lei L., Hou Y. (2018). Nanostructured Ternary Metal Tungstate-Based Photocatalysts
for Environmental Purification and Solar Water Splitting: A Review. Nanomicro Lett..

[21] Kölbach M., Pereira I. J., Harbauer K., Plate P., Höflich K., Berglund S. P., Friedrich D., van de Krol R., Abdi F. F. (2018). Revealing the Performance Limiting
Factors in α-SnWO4
Photoanodes for Solar Water Splitting. Chem.
Mater..

[22] Harb M., Ziani A., Takanabe K. (2016). Critical Difference
between Optoelectronic
Properties of α- and β-SnWO4 Semiconductors: A DFT/HSE06
and Experimental Investigation. Phys. Status
Solidi B Basic Res..

[23] He G., Li J., Qiu W., Chen L., Wang K., Liu Y., Liu M., Li W. (2023). Engineering Surficial Atom Arrangement on α-SnWO4
Film for Efficient Photoelectrochemical Water Splitting. Chem. Eng. J..

[24] Kölbach M., Hempel H., Harbauer K., Schleuning M., Petsiuk A., Höflich K., Deinhart V., Friedrich D., Eichberger R., Abdi F. F., van de Krol R. (2020). Grain Boundaries
Limit the Charge Carrier Transport in Pulsed Laser Deposited α-SnWO4
Thin Film Photoabsorbers. ACS Appl. Energy Mater..

[25] Kong H., Abdi F. F. (2023). Recent Progress
in the Development of Tin Tungstate
(α-SnWO4) Photoanodes for Solar Water Oxidation. Inorg. Chem. Front..

[26] Minohara M., Dobashi Y., Kikuchi N., Samizo A., Tsukuda K., Nishio K., Mibu K., Kumigashira H., Hase I., Yoshida Y., Aiura Y. (2021). Bipolar Semiconducting
Properties in α-SnWO4 Based on the Characteristic Defect Structure. Inorg. Chem..

[27] Gottesman R. (2025). Physical Vapor
Deposition with Rapid Photonic Annealing: Enhanced Stability in Metal
Oxide Photoelectrodes. J. Phys. Chem. C.

[28] Kölbach M., Hempel H., Harbauer K., Schleuning M., Petsiuk A., Höflich K., Deinhart V., Friedrich D., Eichberger R., Abdi F. F., van de Krol R. (2020). Grain Boundaries
Limit the Charge Carrier Transport in Pulsed Laser Deposited α-SnWO4
Thin Film Photoabsorbers. ACS Appl. Energy Mater..

[29] Kölbach M., Pereira I. J., Harbauer K., Plate P., Höflich K., Berglund S. P., Friedrich D., van de Krol R., Abdi F. F. (2018). Revealing the Performance Limiting
Factors in α-SnWO4
Photoanodes for Solar Water Splitting. Chem.
Mater..

[30] Gauzzi F., Verdini B., Maddalena A., Principi G. (1985). X-Ray Diffraction and
Mössbauer Analyses of SnO Disproportionation Products. Inorg. Chim. Acta.

[31] Harb M., Cavallo L., Basset J. M. (2020). Remarkable
Influence of α-SnWO4
Exposed Facets on Their Photocatalytic Performance for H2 and O2 Evolution
Reactions. J. Phys. Chem. C.

[32] Gottesman R., Peracchi I., Gerke J., Irani R., Abdi F. F., van de Krol R. (2022). Shining a
Hot Light on Emerging Photoabsorber Materials:
The Power of Rapid Radiative Heating in Developing Oxide Thin-Film
Photoelectrodes. ACS Energy Lett..

[33] Azofra L. M., Cavallo L., Basset J. M., Harb M. (2021). Need for Rationally
Designed SnWO4 Photo­(Electro)­Catalysts to Overcome the Performance
Limitations for O2 and H2 Evolution Reactions. J. Phys. Chem. C.

[34] Singh R. (1988). Rapid Isothermal
Processing. J. Appl. Phys..

[35] Rebohle L., Prucnal S., Skorupa W. (2016). A Review of
Thermal Processing in
the Subsecond Range: Semiconductors and Beyond. Semicond. Sci. Technol..

[36] Momma K., Izumi F. (2011). VESTA 3 for Three-Dimensional
Visualization of Crystal, Volumetric
and Morphology Data. J. Appl. Crystallogr..

[37] Rettie A. J. E., Chemelewski W. D., Emin D., Mullins C. B. (2016). Unravelling
Small-Polaron Transport in Metal Oxide Photoelectrodes. J. Phys. Chem. Lett..

[38] Bozheyev F., Fengler S., Kollmann J., Abou-ras D., Scharnagl N., Schieda M. (2024). Influence of SnWO4,
SnW3O9, and WO3 Phases in Tin Tungstate
Films on Photoelectrochemical Water Oxidation. ACS Appl. Mater. Interfaces.

[39] Warren S. C., Voïtchovsky K., Dotan H., Leroy C. M., Cornuz M., Stellacci F., Hébert C., Rothschild A., Grätzel M. (2013). Identifying Champion Nanostructures for Solar Water-Splitting. Nat. Mater..

[40] Nagappan S., Minhas H., Urkude R. R., Pathak B., Kundu S. (2025). Harnessing
the Synergistic Effects of Ir Co-Decorated NiMn-LDH: A Multi-Analytical
Approach to Boost Electrocatalytic Overall Water Splitting Activity
in Alkaline Condition. Small.

