# Supplementary material for: Crystallographic Anisotropies in α‑SnWO4 Photoelectrodes and Their Effects on Electronic Properties and Photoelectrochemical Performances
Source: ACS Appl Mater Interfaces. 2026 May 16;18(20):28768–76. doi: 10.1021/acsami.6c04445 (PMC13220226; doi:10.1021/acsami.6c04445)
Supplement: Supplementary file 1 [file am6c04445_si_001.pdf]

Supporting Information

Crystallographic Anisotropies in α-$SnWO_4$ Photoelectrodes and Their Effects on Electronic Properties and Photoelectrochemical Performances

Ronen Gottesman,*[1,2] Erwin Fernandez,[3] Rene Schwiddessen,[4] Daniel Abou-Ras,[4] Doron Azulay,[5,6] Oded Millo,[2,5] Roel van de Krol*[3,7]

[1]The Institute of Chemistry, The Hebrew University of Jerusalem Edmond J. Safra Campus, Givat Ram, Jerusalem 9190401, Israel

[2]The Center for Nanoscience and Nanotechnology, The Hebrew University of Jerusalem Edmond J. Safra Campus, Givat Ram, Jerusalem 9190401, Israel

[3]Institute for Solar Fuels, Helmholtz-Zentrum Berlin für Materialien und Energie GmbH Hahn-Meitner-Platz 1, 14109 Berlin, Germany

[4]Department of Structure and Dynamics of Energy Materials Helmholtz-Zentrum Berlin für Materialien und Energie GmbH Hahn-Meitner-Platz 1, 14109 Berlin, Germany

[5]The Racah Institute of Physics, The Hebrew University of Jerusalem Edmond J. Safra Campus, Givat Ram, Jerusalem 9190401, Israel

[6]Department of Physics, Azrieli College of Engineering, Jerusalem 9103501, Israel.

[7]Institut für Chemie Technische Universität Berlin, Straße des 17. Juni 124, 10623 Berlin, Germany

Corresponding authors: ronen.gottesman@mail.huji.ac.il; roel.vandekrol@helmholtz-berlin.de

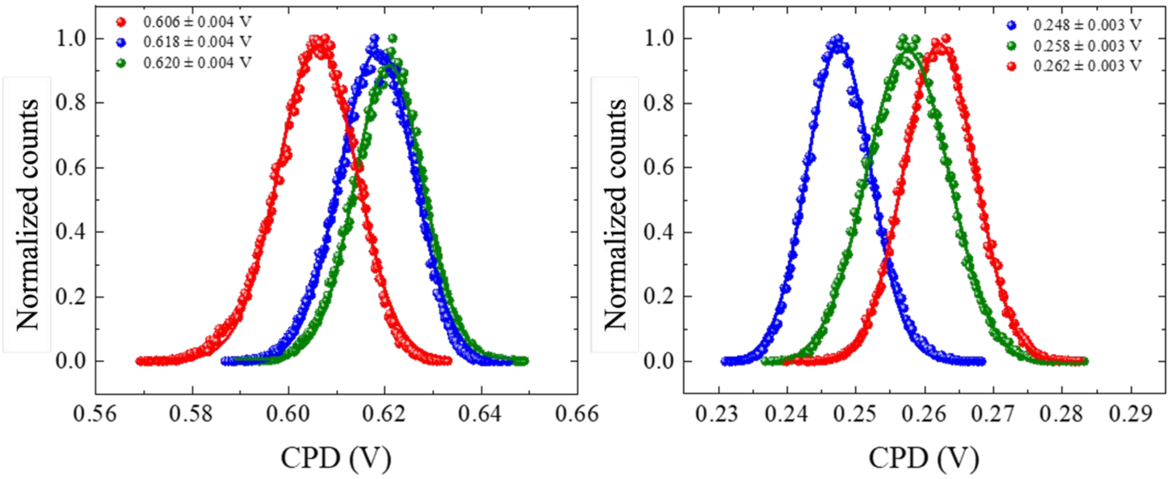

Figure S1. CPD distributions acquired from multiple scan areas on (a) RTP-treated and (b) FH-treated α-$SnWO_4$ films. Each panel shows three independent scan areas with Gaussian fits (solid lines). The mean CPD values and standard deviations extracted from each fit are indicated in the legends (with corresponding colors). The overall mean CPD values across scan areas are 0.256 ± 0.007 V for the RTP-treated film and 0.615 ± 0.008 V for the FH-treated film, confirming the consistency of the CPD difference (~0.35 V) reported in Figure 5.
